# GSK-3β Inhibitor Alsterpaullone Attenuates MPP^+^-Induced Cell Damage in a c-Myc-Dependent Manner in SH-SY5Y Cells

**DOI:** 10.3389/fncel.2018.00283

**Published:** 2018-08-30

**Authors:** Jiancai Wang, Yuqian Li, Li Gao, Fengqi Yan, Guodong Gao, Lihong Li

**Affiliations:** ^1^Department of Neurosurgery, Tangdu Hospital, Fourth Military Medical University, Xi’an, China; ^2^Department of Urology, Tangdu Hospital, Fourth Military Medical University, Xi’an, China

**Keywords:** Parkinson’s disease, mitochondria, wnt signaling, c-Myc, alsterpaullone

## Abstract

Mitochondrial dysfunction plays significant roles in the pathogenesis of Parkinson’s Disease (PD). The inactivation of c-Myc, a down-stream gene of Wnt/β-catenin signaling, may contribute to the mitochondria dysfunction. Inhibition of glycogen synthase kinase 3β (GSK-3β) with Alsterpaullone (Als) can activate the down-stream events of Wnt signaling. Here, we investigated the protective roles of Als against MPP^+^-induced cell apoptosis in SH-SY5Y cells. The data showed that Als effectively rescued c-Myc from the MPP^+^-induced decline via Wnt signaling. Furthermore, Als protected SH-SY5Y cells from the MPP^+^-induced mitochondrial fission and cell apoptosis. However, the protective roles of Als were lost under β-catenin-deficient conditions. These findings indicate that Als, a GSK-3β inhibitor, attenuated the MPP^+^-induced mitochondria-dependent apoptotic via up-regulation of the Wnt signaling.

## Introduction

Parkinson’s Disease (PD) is a common movement disorder characterized by the selective loss of dopaminergic neurons in substania nigra pars compacta (Rostamian Delavar et al., 2018; Sheehan and Yue, 2018). Accumulating evidences have suggested that mitochondrial dysfunction plays important roles in the pathogenesis of PD (Wang et al., 2016; Perez Carrion et al., 2018). However, the underlying molecular mechanisms responsible for mitochondrial dysfunction are elusive and remain to be fully explored. Emerging evidences show that inactivation of c-Myc, a Wnt/β-catenin signaling down-stream gene, may contribute to the fusion of mitochondria (Graves et al., 2012). Besides, the down-regulation of  β-catenin and c-Myc has been reported in 6-OHDA induced animal PD models (Wang et al., 2017). The down-regulation of p-Ser9-glycogen synthase kinase 3β (GSK3β) also has been reported *in vitro* parkinsonism models (Dun et al., 2012).

Wnt signaling modulates various processes in central nervous system development, such as neurogenesis, synapse plasticity and neuronal survival (Lambert et al., 2016; Oliva et al., 2018). β-catenin is one of the main effectors in canonical Wnt signaling (Wang et al., 2017). The enzyme GSK-3β is an inhibitor of canonical Wnt signaling as it leads to the degradation of β-catenin (Rangrez et al., 2016). Inhibition of the GSK-3β activity by molecular compounds and various enzymes is an important step upon activation of the canonical Wnt signaling cascade and the downstream genes expression, including c-Myc and Cyclin D1 (Kouvidi et al., 2016). Alsterpaullone (Als) is demonstrated to act by competing with the ATP for binding to GSK-3β and induce the phosphorylation of GSK-3β on serine-9 (Leost et al., 2000; Teo et al., 2006). Activating downstream events of Wnt signaling by inhibiting GSK-3β with Als induces the recruitment of nerve cells from interstitial stem cells (Teo et al., 2006). Furthermore, the increase of phosphorylated GSK-3β has a crucial role in the prevention of cortical neurons apoptosis (Takadera et al., 2012).

Mitochondria act as an important mediator of the survival and apoptosis in nerve cells. Increasing evidence indicates that the dysfunction of mitochondria plays a crucial role in the pathophysiology of PD (Hu et al., 2014; Monti et al., 2015). Mitochondrial fission often produces small and dysfunctional organelles which is eliminated by autophagosomal machinery, However, the fusion can maintain the integrity of mitochondria and thereby prolonging the mitochondria life spans (Shimauchi et al., 2017). Graves et al. (2012) have reported that c-Myc is responsible for the maintaining of mitochondrial membrane potential and the increasing of membrane fusion.

Wnt/β-catenin signaling plays a vital and direct role in the loss of dopaminergic neurons in PD (Wang et al., 2017). c-Myc, a down-stream gene of Wnt/β-catenin signaling, may modulate the fusion of mitochondria (Graves et al., 2012). Inhibition of GSK-3β with Als can activate the downstream events of Wnt signaling (Takadera et al., 2012). Furthermore, the dysfunction of mitochondria appears to participate in the pathophysiology of PD (Wang et al., 2016). In our study, we investigated the protective effects of Als against the MPP^+^-induced mitochondrial fission and cell apoptosis in SH-SY5Y cells and the role of c-Myc in these protections.

## Materials and Methods

### Reagents

Fetal bovine serum (FBS) and Dulbecco’s modified Eagle’s medium (DMEM) was provided by Gibco (Gai-thersburg, MD, USA). MTT, MPP^+^ and Als were obtained from Sigma-Aldrich (St. Louis, MO, USA). MitoTracker was purchased from Life Technologies (Carlsbad, CA, USA, Cat#1837173). The following antibodies were used: β-catenin (Abcam, Cambridge, UK, Cat#ab19449, RRID:AB_444927), PARP (Abcam, Cambridge, UK, Cat#ab32138, RRID:AB_777101), c-Myc (Abcam, Cambridge, UK, Cat# ab17356, RRID:AB_2148459), GAPDH (Cell Signaling, Beverly, MA, USA, Cat#3683, RRID:AB_1642205), GSK-3β (Cell Signaling, Beverly, MA, USA, Cat#12456, RRID:AB_2636978), p-GSK-3β (ser9; Cell Signaling, Beverly, MA, USA, Cat#9322P, RRID:AB_2115199), cleaved caspase-3 (Cell Signaling, Beverly, MA, USA, Cat#9669, RRID:AB_2069869), cleaved caspase-8 (Cell Signaling, Beverly, MA, USA, Cat#9496L, RRID:AB_2259431), β-actin (Biosynthesis biotechnology, Beijing, china), FITC or TRITC-conjugated secondary antibody (Biosynthesis biotechnology, Beijing, china, Cat#BSTEK021, Cat#BST12B15B31), Anti-mouse-HRP IgG or anti-rabbit -HRP IgG (Biosynthesis biotechnology, Beijing, china, Cat#RS0002, Cat#ZB2305).

### Cell Cultures

SH-SY5Y cells, a human dopaminergic neuroblastoma cell line, were purchased from ATCC (Manassas, VA, USA, Cat#CRL-2266, RRID:CVCL_0019). Then the cells were cultured in DMEM supplemented with 10% FBS in a humidified atmosphere of 5% CO2 at 33°C. Cells were incubated with a range of MPP^+^ concentrations (0–2,000 μM) for 24 h. Various concentrations of Als (0–2.0 μM) were added for 24 h before MPP^+^ treatment.

### Gene Transfection

Human β-catenin in the pcDNA3.0 vector was purchased from Addgene (#16828, Cambridge, MA, USA). The β-catenin small interfering RNA and c-Myc small interfering RNA were obtained from GenePharma (Shanghai, China). SH-SY5Y cells were seeded in the 6-well plates and cultured in DMEM without FBS. Then the cells were transfected with 60 nM siRNA or the indicated plasmids using Lipofectamin 2,000 according to the manufacturer’s instructions. Cells were exposed to the plasmid or siRNA for 6 h, after which the medium was replaced by complete DMEM. The siRNAs sequences are shown in the Supplementary Table S1.

### MTT Assay

Cell viability was measured by MTT assay according to the protocols. Briefly, cells grown in 96-well microplates were incubated with MTT labeling reagents for 2 h at 37°C. Then the supernate was removed and 100 μl DMSO was added to each well (Zeng et al., 2017). The absorbance at 490 nm was determined by microplates reader (Bio-Rad, Hercules, CA, USA).

### Western Blot Analysis

Total proteins were prepared from cell lines. The equal amounts of proteins were separated by SDS-PAGE and transferred to the polyvinylidene fluoride membranes. The membranes were blocked in 5% BSA at room temperature for 2 h and incubated with primary antibodies at 4°C overnight. After washing three times, membranes were incubated with the HRP-conjugated secondary antibody at room temperature for 2 h. Chemiluminescence detection was used to visualize the interest protein bands.

### Nuclear Extraction

Cells were transferred to the 1.5 ml microcentrifuge tube and centrifuge at 3,000 rpm for 5 min at 4°C. A pipette was used to carefully discard the supernatant. 0.6 ml hypotonic buffer was added to the microcentrifuge tube. Cell pellet in the microcentrifuge tube was suspended vigorously and incubated on ice for 10 min. Then the tube was centrifuged at 3,000 rpm for 5 min at 4°C and the supernatant was removed. The precipitate was suspended vigorously after 0.2 ml lysis buffer was added. The tube was incubated on ice for 20 min and centrifuged at 15,000 rpm for 5 min at 4°C. The supernatant was nuclear extraction of SH-SY5Y cells.

### Quantitative Real-Time PCR

The total RNA was isolated from SH-SY5Y cells using Trizol Reagent (Invitrogen), and then cDNA was synthesized using the AMV reverse transcriptase (Promega, Madison, WI, USA). The quantitative real-time PCR was conducted using ABI Prism 7,500 real-time PCR instrument (Applied Biosystems, Carlsbad, CA, USA). The primer sequences are shown in Supplementary Table S2.

### Immunofluorescence

Cells on the slides were washed with PBS and fixed with 4% formaldehyde solution for 8 min. After permeabilizing for 30 min in the 0.1% Triton-100, the cells were blocked with 1% BSA for 2 h and incubated with primary antibodies at 4°C overnight. The next day, cells were washed with PBS for three times and labeled with secondary antibodies. Then the nuclei were labeled with DAPI for 15 min at room temperature. Nikon fluorescence microscope (C2 Si; Nikon, Japan) was used to capture the fluorescence images.

### Mitochondrial Staining

Mitochondrial staining was performed according to the manufacturer’s instructions. Briefly, SH-SY5Y cells were grown on a confocal Petri dish. After designed treatment, they were stained with 10 nM MitoTracker Red CMXRos (Life Technologies) for 20 min at 33°C. Then the cells were fixed with 4% formaldehyde solution for 8 min and labeled with DAPI for 15 min at room temperature. Nikon fluorescence microscope (C2 Si; Nikon, Japan) was used to observe the mitochondria. The ratio between the major and minor axes of an ellipse equivalent to the shape of the mitochondrial is regard as the aspect ratio of mitochondria. Twenty fields of view in a coverslip are randomly recorded to analyze the aspect ratio of mitochondria in an experiment (Koopman et al., 2008; Zeng et al., 2017).

### TUNEL Assay

TUNEL assay was conducted using the one step TUNEL Apoptosis Kit (Beyotime, JS, China). After treatment, cells plated in confocal Petri dishes were fixed and incubated with TUNEL reaction buffer at 37°C for 1 h according to the manufacture’s protocol. Then the cells were washed with PBS for three times and stained with DAPI. The TUNEL positive cells were imaged under a Nikon fluorescence microscope (C2 Si; Nikon, Japan).

### Statistical Analysis

Statistical difference was measured using one-way analysis of variance (ANOVA) followed by Bonferroni test for multiple groups. All values were reported as the mean ± SEM. *P* values < 0.05 were considered to be statistically significant.

## Results

### Effects of Als on MPP^+^-Induced Neurotoxicity and Inactivation of Wnt Signaling in SH-SY5Y Cells

**Figure 1:**
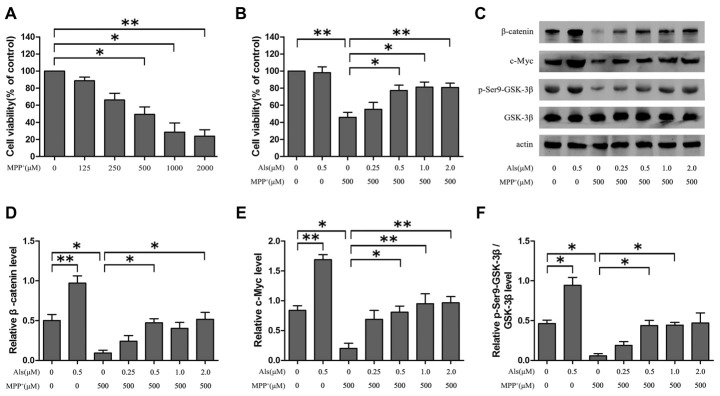
The effect of Alsterpaullone (Als) against MPP^+^-induced cytotoxicity and inactivation of Wnt signaling. **(A)** Cells were treated with a range of MPP^+^ concentrations (0–2,000 μM) for 24 h, and MTT assay was used to detect the cell viability. **(B)** Various concentrations of Als (0–2.0 μM) were added for 24 h before MPP^+^ treatment (500 μM). Cell viability was evaluated using the MTT assay (the means ± SEM; *n* = 3; *p < 0.05, **p < 0.01). **(C)** Cells were treated as described in **(B)** and Western blot was conducted in the different groups. **(D–F)** Relative amounts of β-catenin/actin, c-Myc/actin and p-glycogen synthase kinase 3β (GSK-3β; ser9)/GSK-3β were analyzed (the means ± SEM; *n* = 3; *p < 0.05, **p < 0.01).

Cells were treated with a series of concentrations (0, 125, 250, 500, 1,000, 2,000 μM) of MPP^+^ for 24 h. The MTT assay indicated that MPP^+^ induced a dose-dependent decrease of the cell viability, and the cell viability decreased almost 45% at an exposure to 500 μM MPP^+^ (Figure 1A). However, the cell viability of SH-SY5Y cells exhibited an increase when cells were pretreated with various concentrations of Als (0.5, 1.0, 2.0 μM) for 24 h before MPP^+^= treatment (Figure 1B). Moreover, Als (0.25 μM or 0.5 μM) was applied to SH-SY5Y cells treated with MPP^+^ and the cell viability was assessed 0 h, 12 h, 24 h or 48 h later. The results indicated that the decrease of cell viability induced by MPP^+^ could be abrogated by 0.5 μM and 24 h Als treatment (Supplementary Figure S1). Although 0.5 μM Als treatment alone showed no difference on the cell viability compared to that of the control group, we still found an activation of Wnt signaling in 0.5 μM Als treatment alone group compared with control group (Figures 1B,C). Besides, the down-regulation of Wnt signaling induced by MPP^+^ (500 μM) could be abrogated by pretreatment of Als (0.5, 1.0, 2.0 μM; Figures 1C–F). Then the cells were pretreated with 0.5 μM Als for 24 h before adding MPP^+^ in the following experiments. Furthermore, these results indicated that Als could protect SH-SY5Y cells from the MPP^+^-induced down-regulation of cell viability and Wnt signaling in a dose-dependent manner.

### Als Rescued c-Myc From the MPP^+^-Induced Decline via Wnt Signaling

**Figure 2:**
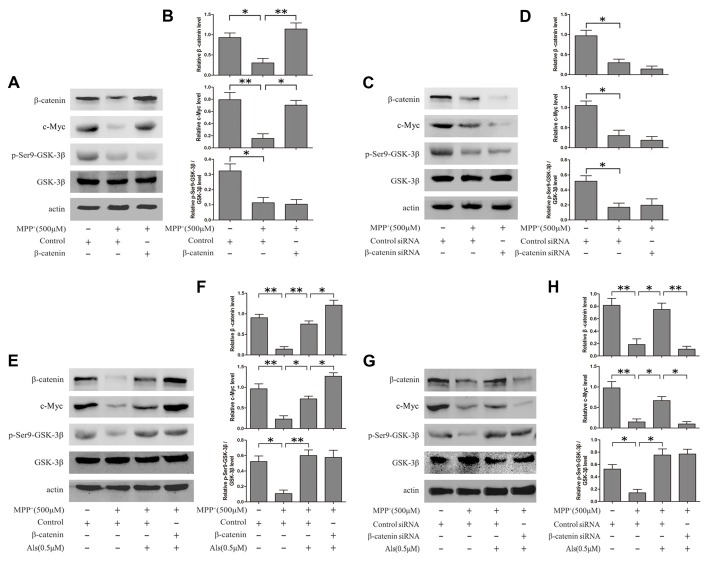
Als effectively rescued c-Myc from the MPP^+^-induced decline via Wnt signaling. **(A)** Cells transfected with pcDNA3.0-β-catenin were treated with MPP^+^ (500 μM) for 24 h. Western blot was conducted in the Control, MPP^+^ and MPP^+^+β-catenin groups. **(B)** Relative amounts of β-catenin/actin, c-Myc/actin and p- GSK-3β (ser9)/GSK-3β level were quantified (the means ± SEM; *n* = 3; *p < 0.05, **p < 0.01). **(C)** Cells transfected with β-catenin siRNA were treated with MPP^+^ (500 μM) for 24 h. Western blot was conducted in the Control siRNA, MPP^+^ and β-catenin siRNA groups. **(D)** The ratios of β-catenin/actin, c-Myc/actin and p-GSK-3β(ser9)/GSK-3β were analyzed (the means ± SEM; *n* = 3; *p < 0.05, **p < 0.01). **(E)** Cells transfected with pcDNA3.0 or pcDNA3.0-β-catenin were pretreated with Als for 24 h before MPP^+^ treatment. Western blot was conducted in different groups. **(F)** Quantification graphs of the ratios of β-catenin/actin, c-Myc/actin and p-GSK-3β (ser9)/GSK-3β (the means ± SEM; *n* = 3; *p < 0.05, **p < 0.01). **(G)** Cells transfected with control siRNA or β-catenin siRNA were pretreated with Als for 24 h before MPP^+^ treatment and Western blot was conducted in different groups. **(H)** Quantification graphs indicating the ratios of β-catenin/actin, c-Myc/actin and p-GSK-3β(ser9)/GSK-3β (the means ± SEM; *n* = 3; *p < 0.05, **p < 0.01).

The expression levels of c-Myc and Wnt signaling were analyzed by western blotting. The results showed that MPP^+^ treatment led to the down-regulation of c-Myc and Wnt signaling. Then the β-catenin was overexpressed using pcDNA3.0-β-catenin in SH-SY5Y cells (Supplementary Figure S2). Overexpression of β-catenin could reverse the inactivation of c-Myc induced by MPP^+^ treatment (Figures 2A–D). Besides, Als attenuated the MPP^+^-induced down-regulation of c-Myc and Wnt signaling. Overexpression of β-catenin exacerbated the increase of c-Myc in SH-SY5Y cells pretreated with Als before adding MPP^+^ (Figures 2E,F). Moreover, Als could reverse the down-regulation of c-Myc induced by the adding of MPP^+^ and silencing β-catenin largely abrogated this positive effect of Als in SH-SY5Y cells (Figures 2G,H, Supplementary Figure S3).

**Figure 3:**
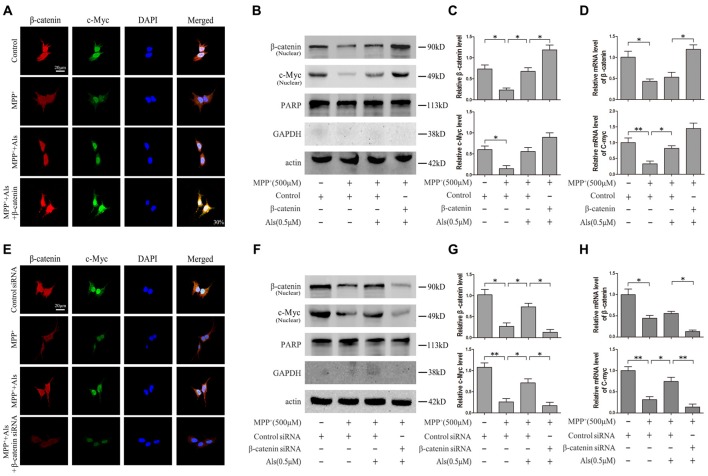
Expression of c-Myc and β-catenin in the nuclear fractions and mRNA levels of SH-SY5Y cells. **(A)** Immunofluorescence analysis of c-Myc and β-catenin in SH-SY5Y cells. Cells were treated as described in Figure 2E (scale bar, 20 μm; Transfection efficiency, 30%). **(B)** Western blot analysis of the nuclear fractions of SH-SY5Y cells which were treated as described in Figure 2E. GAPDH and PARP was the markers for the cytoplasmic and nuclear fractions, respectively. **(C)** Quantification graphs of the β-catenin/actin and c-Myc/actin in the nuclear fractions (the means ± SEM; *n* = 3; *p < 0.05). **(D)** The mRNA level of β-catenin and c-Myc in different groups of SH-SY5Y cells, and actin served as the internal control to ensure equal loading (the means ± SEM; *n* = 3; *p < 0.05, **p < 0.01). **(E)** Immunofluorescence analysis of c-Myc and β-catenin in SH-SY5Y cells which were treated as described in Figure 2G (scale bar, 20 μM). **(F)** Western blot analysis of the nuclear fractions of SH-SY5Y cells which were treated as described in Figure 2G. **(G)** The ratios of β-catenin/actin and c-Myc/actin were analyzed (the means ± SEM; *n* = 3; *p < 0.05, **p < 0.01). **(H)** The mRNA level of β-catenin and c-Myc in different groups of SH-SY5Y cells, and actin served as the internal control to ensure equal loading (the means ± SEM; *n* = 3; *p < 0.05, **p < 0.01).

To analyze the level of c-Myc and β-catenin in nuclear fraction, we performed the immunofluorescence. The results indicated that Als attenuated the MPP^+^-induced down-regulation of c-Myc and β-catenin and the increase of c-Myc could be exacerbated by the overexpression of β-catenin in the nuclear fraction of SH-SY5Y cells (Figure 3A). Western blotting results also revealed that Als attenuated MPP^+^-induced down-regulation of c-Myc and β-catenin in the nuclear fraction of SH-SY5Y cells (Figures 3B,C). The mRNA level of c-Myc and β-catenin decreased after MPP^+^ treatment and Als could reverse the decrease of c-Myc mRNA induced by MPP^+^ (Figure 3D). Then the immunofluorescence showed that Als reversed the down-regulation of c-Myc induced by MPP^+^ and silencing β-catenin largely abrogated this positive effect of Als in the nuclear fraction of SH-SY5Ycells (Figure 3E). Same conclusion was obtained in the western blotting of the nuclear fraction and quantitative real-time PCR test (Figures 3F–H).

### Als Protected SH-SY5Y Cells From the MPP^+^-Induced Mitochondrial Fission via c-Myc

**Figure 4:**
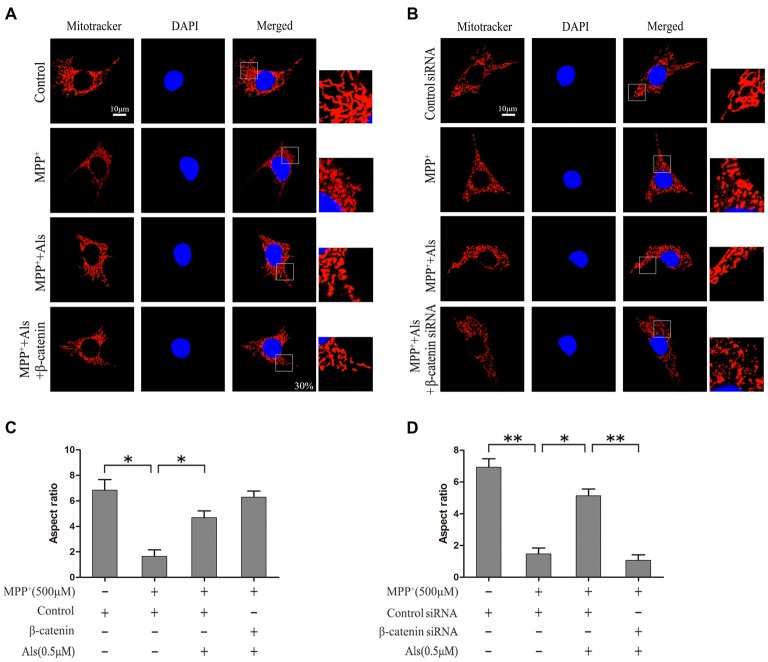
Protection of Als against the MPP^+^-induced mitochondrial fission in SH-SY5Y cells. **(A)** Cells were treated as described in Figure 2E. The morphology of mitochondrial was imaged using a confocal microscope (scale bar, 10 μm; Transfection efficiency, 30%). **(B)** Cells were treated as described in Figure 2G. Mitochondrial morphology was captured using a confocal microscope. **(C,D)** Quantification of the morphology of mitochondria. The ratio between the major and minor axes of an ellipse equivalent to the shape of the mitochondrial is regard as the aspect ratio of mitochondria. Twenty fields of view in a coverslip are randomly recorded to analyze the aspect ratio of mitochondria. The experiments were independently taken three times (the means ± SEM; *n* = 3; *p < 0.05, **p < 0.01).

The β-catenin-overexpressing or β-catenin-knockdown SH-SY5Y cells were pretreated with 0.5 μM Als for 24 h before adding MPP^+^. The level of mitochondrial fission and fusion was determined using MitoTracker (Figures 4A,B). The results showed that MPP^+^ induced significant mitochondrial fission. After the pretreatment of Als for 24 before adding MPP^+^, the mitochondrial fission caused by MPP^+^ was partially alleviated (Figure 4C). Conversely, knockdown of β-catenin largely abrogated the protective effect of Als against the mitochondrial fission phenotype induced by MPP^+^ treatment (Figure 4D).

LiCl, an inhibitor of GSK-3β, was often used to investigate the beneficial role of Wnt signaling. In the present study, both Als and LiCl could reverse the decrease of β-catenin protein induced by MPP^+^. However, Als was more effective than LiCl in the protection of mitochondrial fission caused by MPP^+^. The protective effect of Als against the mitochondrial fission phenotype induced by MPP^+^ could also be abrogated by knockdown of c-Myc (Supplementary Figures S3, S4).

### MPP^+^ Induced SH-SY5Y Cell Apoptosis Could Be Prevented by Als via Wnt Signaling

**Figure 5:**
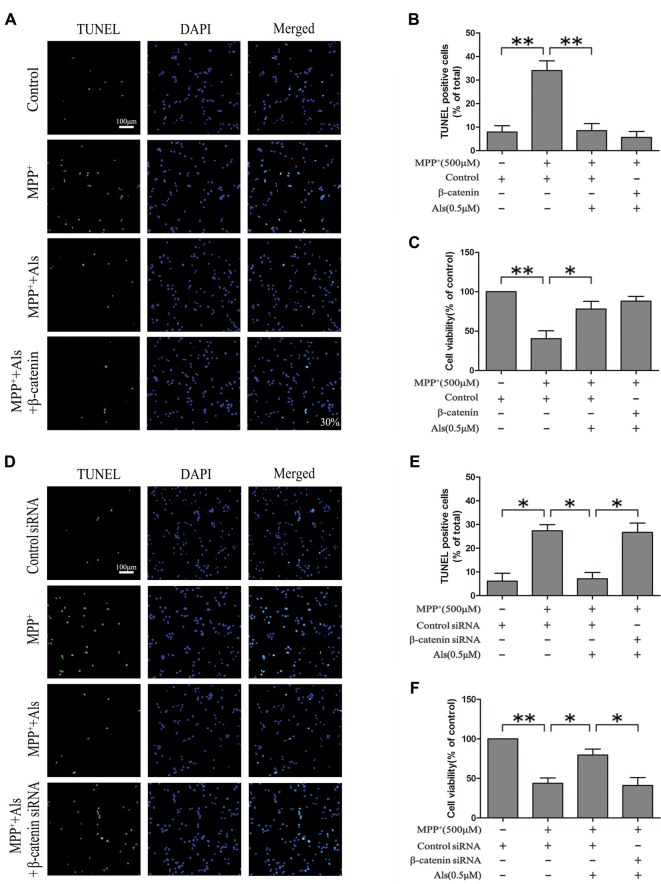
Modulation of the SH-SY5Y cell apoptosis by Als via the Wnt signaling. **(A)** SH-SY5Y cells treated as in Figure 2E were stained for TUNEL (scale bar, 100 μm; Transfection efficiency, 30%). **(B)** The ratios of TUNEL-positive cells/total cells were analyzed (the means ± SEM; *n* = 3; **p < 0.01). **(C)** Cells were treated as described in **(A)** and the cell viability was detected by the MTT assay (the means ± SEM; *n* = 3; *p < 0.05, **p < 0.01). **(D)** Cells treated as described in Figure 2G were assessed for cell apoptosis by TUNEL staining. **(E)** Quantification graphs of the ratios of TUNEL-positive cells/total cells (the means ± SEM; *n* = 3; *p < 0.05). **(F)** The MTT assay was conducted in cells treated as described in (**D**; the means ± SEM; *n* = 3; *p < 0.05, **p < 0.01).

The effect of Als on the MPP^+^ induced cell apoptosis was evaluated by TUNEL staining, cell viability, cleaved caspase-3 level and cleaved caspase-8 level. The results indicated that MPP^+^ treatment induced the increase of TUNEL-positive cells. The pretreatment of Als before adding MPP^+^ caused a significantly decrease in the number of TUNEL-positive SH-SY5Y cells compared with MPP^+^ treatment group (Figures 5A,B). These findings were also supported by the results of cell viability (Figure 5C). However, knockdown of β-catenin abolished the protective effects of Als against MPP^+^-induced cell apoptosis in SH-SY5Y cells, as indicated by the change of TUNEL-positive cells and cell viability (Figures 5D–F).

**Figure 6:**
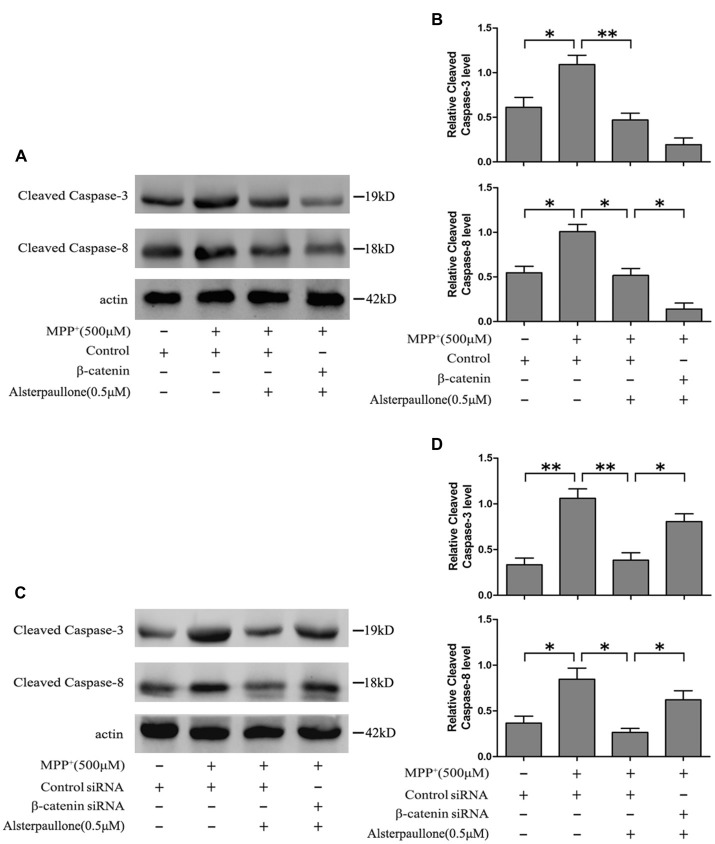
Als attenuated MPP+-induced mitochondrial-mediated cell apoptosis via Wnt signaling. **(A)** Western blot of cleaved-caspase-3, 8 expression in cells treated as described in Figure 2E. **(B)** The relative optical density of cleaved-caspase-3, 8 normalized to actin was analyzed (the means ± SEM; *n* = 3; *p < 0.05, **p < 0.01). **(C)** Cells were treated as described in Figure 2G and Western blot of cleaved-caspase-3, 8 expression was conducted. **(D)** The expression of cleaved-caspase-3, 8 was analyzed (the means ± SEM; *n* = 3; *p < 0.05, **p < 0.01).

Western blotting revealed that MPP^+^ treatment markedly increased cleaved caspase-3 and cleaved caspase-8 expression in the SH-SY5Y cells. Als pretreatment ameliorated the activation of cleaved caspase-3 and cleaved caspase-8 induced by MPP^+^ treatment. Furthermore, the expression of cleaved caspase-8 was decreased in β-catenin overexpression SH-SY5Y cells pretreated with Als before adding MPP^+^ compared with cells pretreated with Als before adding MPP^+^ (Figures 6A,B). However, silencing β-catenin largely abrogated this positive effect of Als against MPP^+^-induced up-regulation of cleaved caspase-3 and cleaved caspase-8 (Figures 6C,D, Supplementary Figure S5).

## Discussion

The loss of dopaminergic neurons is an important pathological feature in the progression of PD (Kaasinen and Vahlberg, 2017; Fox et al., 2018). At present the main objective of PD research is to identify a disease-modifying agent to alleviate the degeneration of dopaminergic neurons (Kim et al., 2016). It has been described that Wnt signaling is associated with the pathogenesis of PD *in vivo* and *in vitro* (Filatova et al., 2011; Dun et al., 2012; Zhang et al., 2016). Previous studies have shown that knockdown of Dkk1, a protein that can inhibit Wnt signaling, exhibited a neuroprotective effect in PC12 cells treated with MPP^+^ (Dun et al., 2013). The neuroprotective role of Wnt signaling in the rat PD model also has been studied (Wang Y.-L. et al., 2017).

GSK-3β is sequestered in cytoplasm fraction and targets β-catenin for ubiquitination and phosphorylation (Oh et al., 2017). Inhibition of GSK-3β function in the ventral midbrain of rats may contribute to the increase of β-catenin level (Dun et al., 2012). In the current study, western blot showed that Als, a GSK-3β inhibitor, attenuated the MPP^+^-induced down-regulation of c-Myc and Wnt signaling. Besides, Als alleviated the SH-SY5Y cell apoptosis induced by MPP^+^ treatment, as indicated by the change of cell viability and TUNEL-positive SH-SY5Y cells. Als also inhibits Cyclin-dependent kinase 5 (CDK5) which has significant role in the pathogenesis of PD (Wen et al., 2014). However, knockdown of β-catenin or c-Myc abolished the protective effects of Als against MPP^+^-induced cell apoptosis in SH-SY5Y cells. These results indicated that the neuroprotective effect of Als may mainly require the activation of Wnt signaling.

Mitochondria are important intercellular organelles in the nerve cells, producing energy through the mitochondrial respiratory chain and acting as a mediator of the cell survival and death (Keilhoff et al., 2016; Mendivil-Perez et al., 2016). Accumulating studies of PD animals and PD patients suggested that mitochondrial dysfunction may play crucial roles in the PD pathogenesis (Martin, 2006; Desmet et al., 2017). The fission of mitochondria produces dysfunctional organelles which can be eliminated by autophagosomal machinery. Fusion, however, is partially effective at preserving the integrity of mitochondria (Kawano et al., 1995; Graves et al., 2012). The mitochondrial abundance may be regulated by the activation of Wnt signaling (Bernkopf et al., 2018). c-Myc is an important downstream genes of Wnt signaling (Hamdoun et al., 2017). The depletion of c-Myc is associated with a rapid decrease in the mitochondrial structural integrity and function. In contrast, Overexpression of c-Myc restores the mitochondrial volume and induces an increased fusion activity (Graves et al., 2012). Then we propose that c-Myc serves to link the Wnt signaling with mitochondrial function and biogenesis via the regulation of fusion and fission processes. Als ameliorated the MPP^+^-induced depletion of c-Myc and this effect could be abrogated by the knockdown of β-catenin. Furthermore, Als alleviated MPP^+^-induced mitochondrial fission in SH-SY5Y cells. Knockdown of β-catenin or c-Myc largely abrogated the protection effect of Als against the mitochondrial dysfunction induced by the adding of MPP^+^. In consideration of the roles of c-Myc on regulation mitochondrial fusion and fission processes, we hypothesize that Als protects the SH-SY5Y cells from the MPP^+^-induced mitochondrial fission in a β-catenin- c-Myc-dependent manner.

Cleaved caspase-3 and cleaved caspase-8 expression have been shown to relate with the mitochondria-dependent apoptotic pathway (Zhang et al., 2013; Shih et al., 2017). In the present study, the activation of caspase-3 and caspase-8 induced by MPP^+^ could be prevented by the pretreatment of Als. Besides, Als lost its protective effect against MPP^+^-induced activation of caspase-3 and caspase-8 when β-catenin was knocked down in SH-SY5Y cells, indicating that Als alleviated the MPP^+^-induced mitochondria-dependent apoptosis via Wnt signaling.

Together with the results presented in our study, the findings indicate that Wnt signaling is a point of the pathogenesis of PD. The Wnt signaling downstream gene c-Myc directly regulates the mitochondria morphology and function. Als, a GSK-3β inhibitor, attenuates the MPP^+^-induced mitochondria-dependent apoptotic via activation of the Wnt signaling. Our study provides potential therapeutic implications of PD as well as the underlying molecular mechanisms of the neuroprotective effect of Als. Considering the complexity of the pathogenesis of PD, further studies should be conducted to elucidate the accurate mechanism.

## Author Contributions

JW and LL designed the study. LG performed the mitochondrial staining and TUNEL assay. YL carried out the immunofluorescence and western blots. The cell culture was conducted by FY. GG and LL wrote the manuscript.

## Conflict of Interest Statement

The authors declare that the research was conducted in the absence of any commercial or financial relationships that could be construed as a potential conflict of interest.

## Abbreviations

Als: Alsterpaullone
CDK5: Cyclin-dependent kinase 5
DMEM: Dulbecco’s modified Eagle’s medium
FBS: Fetal bovine serum
GSK-3β: glycogen synthase kinase 3β
PD: Parkinson’s Disease.
