# Supplementary material for: GSK-3β Inhibitor Alsterpaullone Attenuates MPP+-Induced Cell Damage in a c-Myc-Dependent Manner in SH-SY5Y Cells
Source: Front Cell Neurosci. 2018 Aug 30;12:283. doi: 10.3389/fncel.2018.00283 (PMC6127625; doi:10.3389/fncel.2018.00283)
Supplement: Supplementary file 1 [file Data_Sheet_1.doc]

**Supplementary materials:**

**Supplementary Table 1:** The siRNA sequences targeting β-catenin and c-Myc

| Negative Control siRNA | Sense 5'-UUCUCCGAACGUGUCACGUTT-3' |
| --- | --- |
|  | Antisense 5'-ACGUGACACGUUCGGAGAATT-3' |
| β-catenin siRNA1 | Sense 5'-AGCUGAUAUUGAUGGACAGTT-3' |
|  | Antisense 5'-CUGUCCAUCAAUAUCAGCUTT-3' |
| β-catenin siRNA2 | Sense 5'-AAGUCCUGUAUGAGUGGGAACTT-3' |
|  | Antisense 5'-GUUCCCACUCAUACAGGACUUTT-3' |
| c-Myc siRNA | Sense 5'- CAUCAUCAUCCAGGACUGUAUTT -3' |
|  | Antisense 5'- AUACAGUCCUGGAUGAUGAUGTT -3' |

**Supplementary Table 2:** The primers for real time PCR

| Factors | Primers |
| --- | --- |
| c-Myc | Forward 5'- ACCACCAGCAGCGACTCTGA-3′ |
|  | Reverse 5'- TCCAGCAGAAGGTGATCCAGACT-3′ |
| β-catenin | Forward 5'- GCTTTCAGTTGAGCTGACCA -3′ |
|  | Reverse 5'- CAAGTCCAAGATCAGCAGTCTC-3′ |
| β-actin | Forward 5'- CGCGAGAAGATGACCCAGAT-3' |
|  | Reverse 5'- GTACGGCCAGAGGCGTACAG-3' |


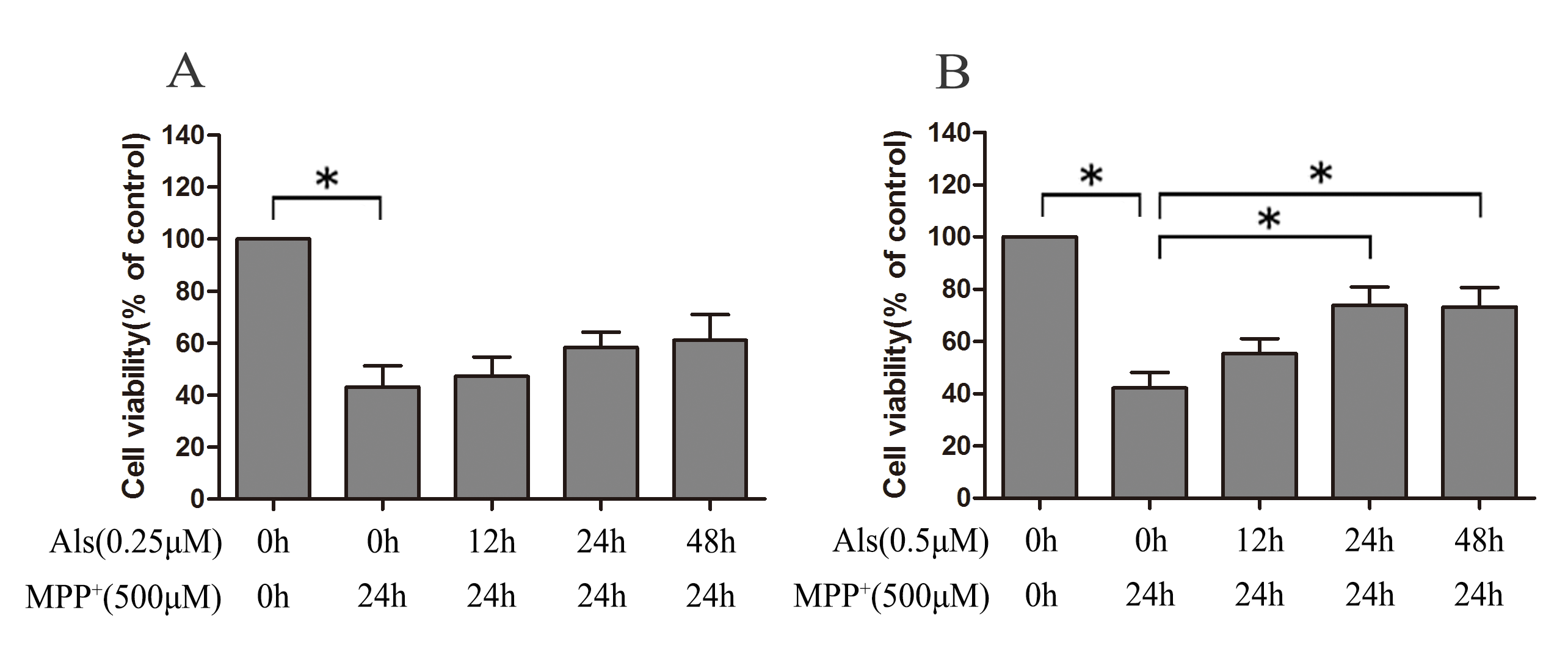


**Supplementary Figure.1:** The effect of different treatment times of Als on MPP^+^-induced cytotoxicity. A: The cells were exposed to MPP^+^(500μM) for 24h and pretreated with Als (0.25μM) for 12h, 24h and 48h before adding MPP^+^. MTT assay was conducted in different groups (the means±SEM; n=3; *p<0.05). B: The cells were pretreated with Als (0.5μM) for 12h, 24h and 48h before MPP^+^ treatment(500μM) (the means±SEM; n=3; *p<0.05).


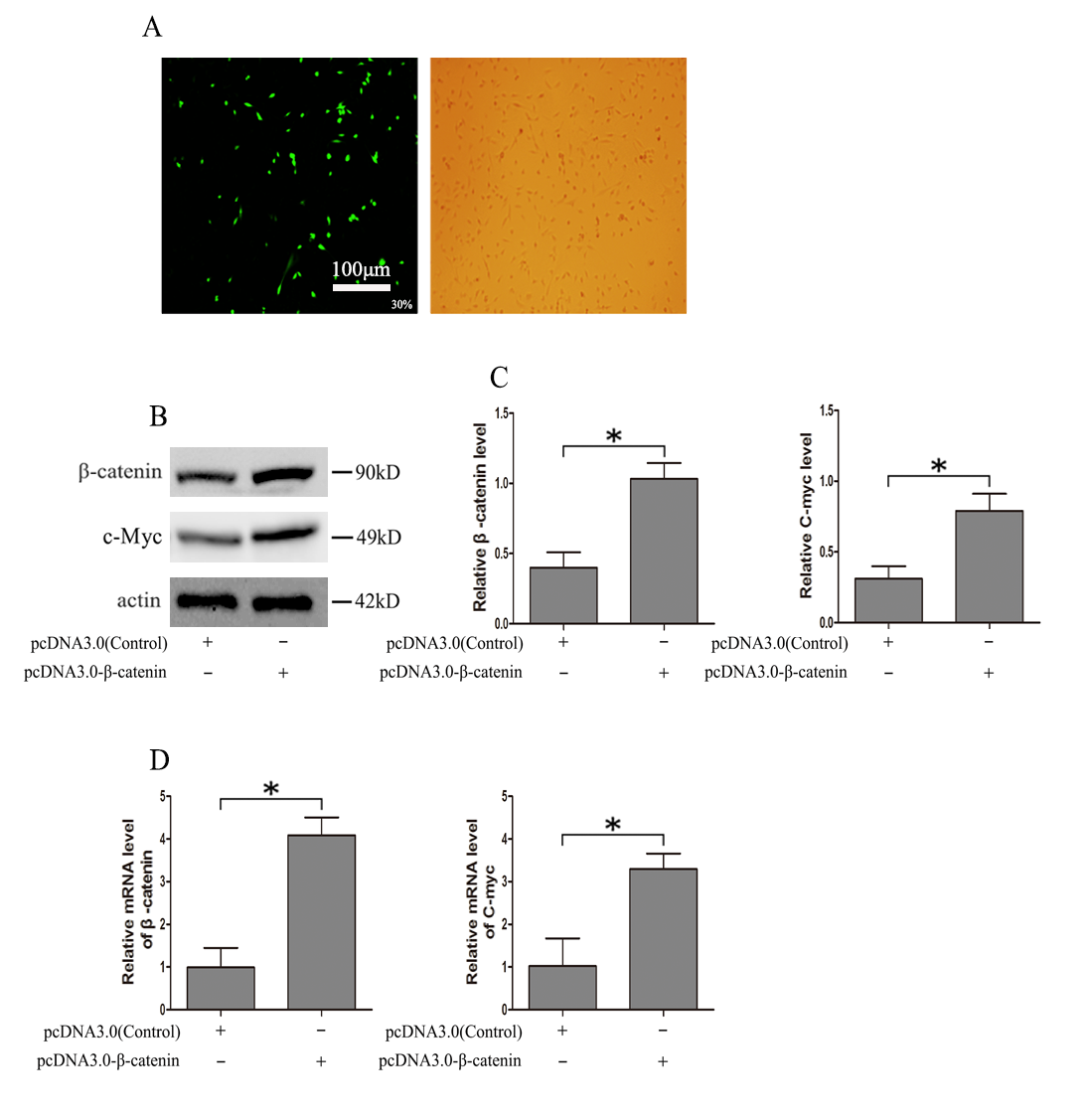


**Supplementary Figure.2:** The overexpression of β-catenin in SH-SY5Y cells. A: Transfection efficiency of pcDNA3.0-β-catenin in SH-SY5Y cells assayed by the GFP plasmid was 30%. B: Cells were transfected with pcDNA3.0 or pcDNA3.0-β-catenin for 48h. C: The ratios of β-catenin/actin and c-Myc/actin were analyzed(the means±SEM; n=3; *p<0.05). D: The mRNA level of β-catenin and c-Myc in SH-SY5Y cells transfected with pcDNA3.0 or pcDNA3.0-β-catenin, and actin served as the internal control to ensure enqual loading(the means±SEM; n=3; *p<0.05).


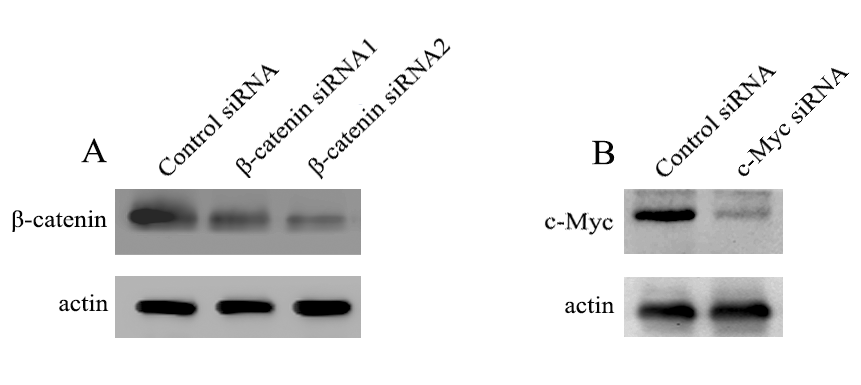


**Supplementary Figure.3:** The SH-SY5Y cells were transfected with siRNAs targeting β-catenin or c-Myc for 48h, after which samples were collected for Western blot. Then the β-catenin siRNA2 and c-Myc siRNA were used in the subsequent studies. (A,B) The down-regulation of β-catenin and c-Myc protein level.


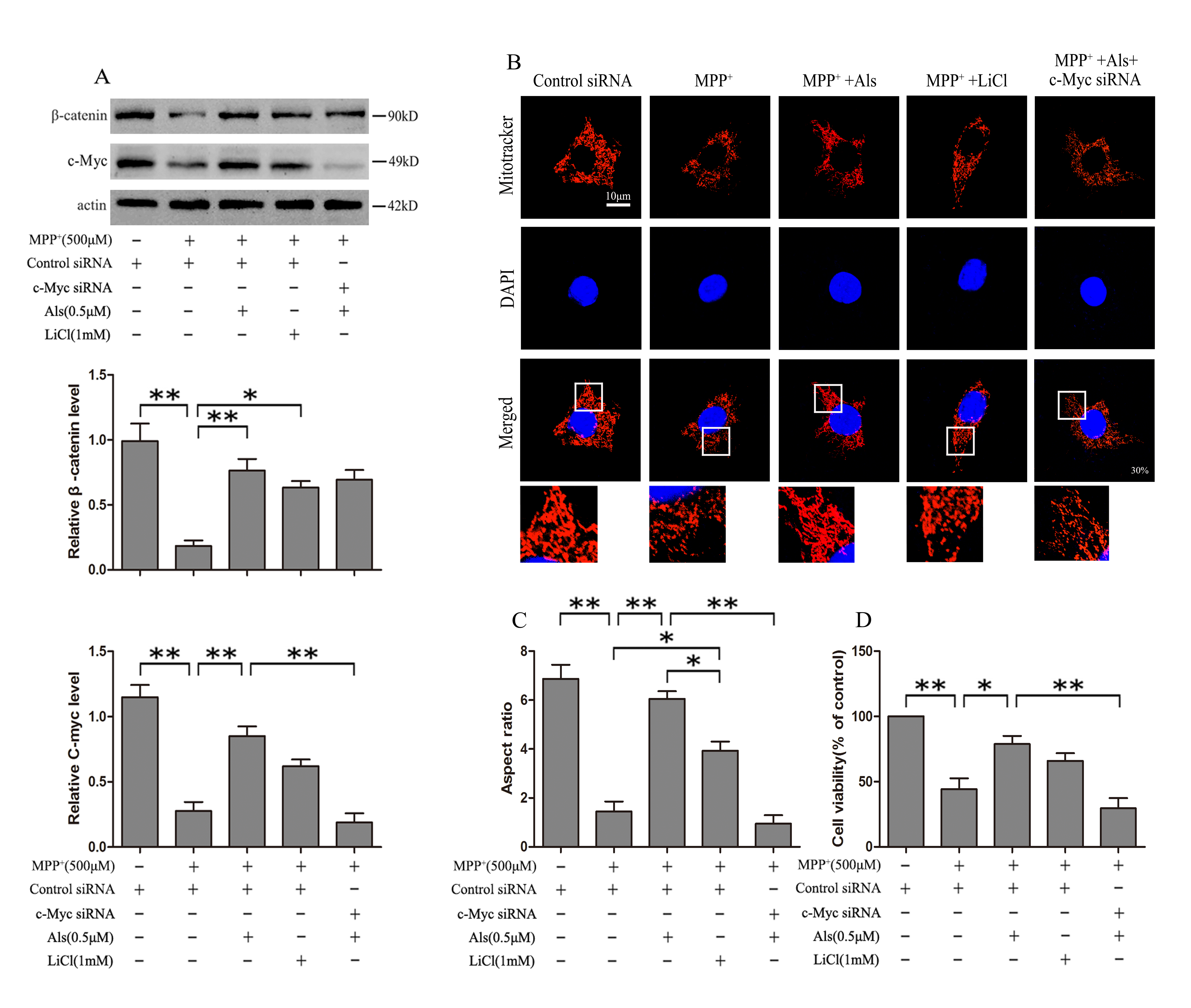


**Supplementary Figure.4:** The effects of LiCl and knockdown of c-Myc in the progress of mitochondria-dependent apoptotic in SH-SY5Y cells induced by MPP^+^.A: Cells transfected with control siRNA or c-Myc siRNA were pretreated with Als or LiCl for 24h before MPP^+^ treatment and Western blot was conducted in different groups. Quantification graphs of the ratios of β-catenin/actin, c-Myc/actin were showed (the means±SEM; n=3; *p<0.05, **p<0.01). B: Cells were treated as described in A. The morphology of mitochondrial was imaged using a confocal microscope(scale bar, 10μm；Transfection efficiency, 30%). C: Quantification of the morphology of mitochondria(the means±SEM; n=3; *p<0.05, **p<0.01). D: The MTT assay was conducted in cells treated as described in A(the means±SEM; n=3; *p<0.05, **p<0.01).


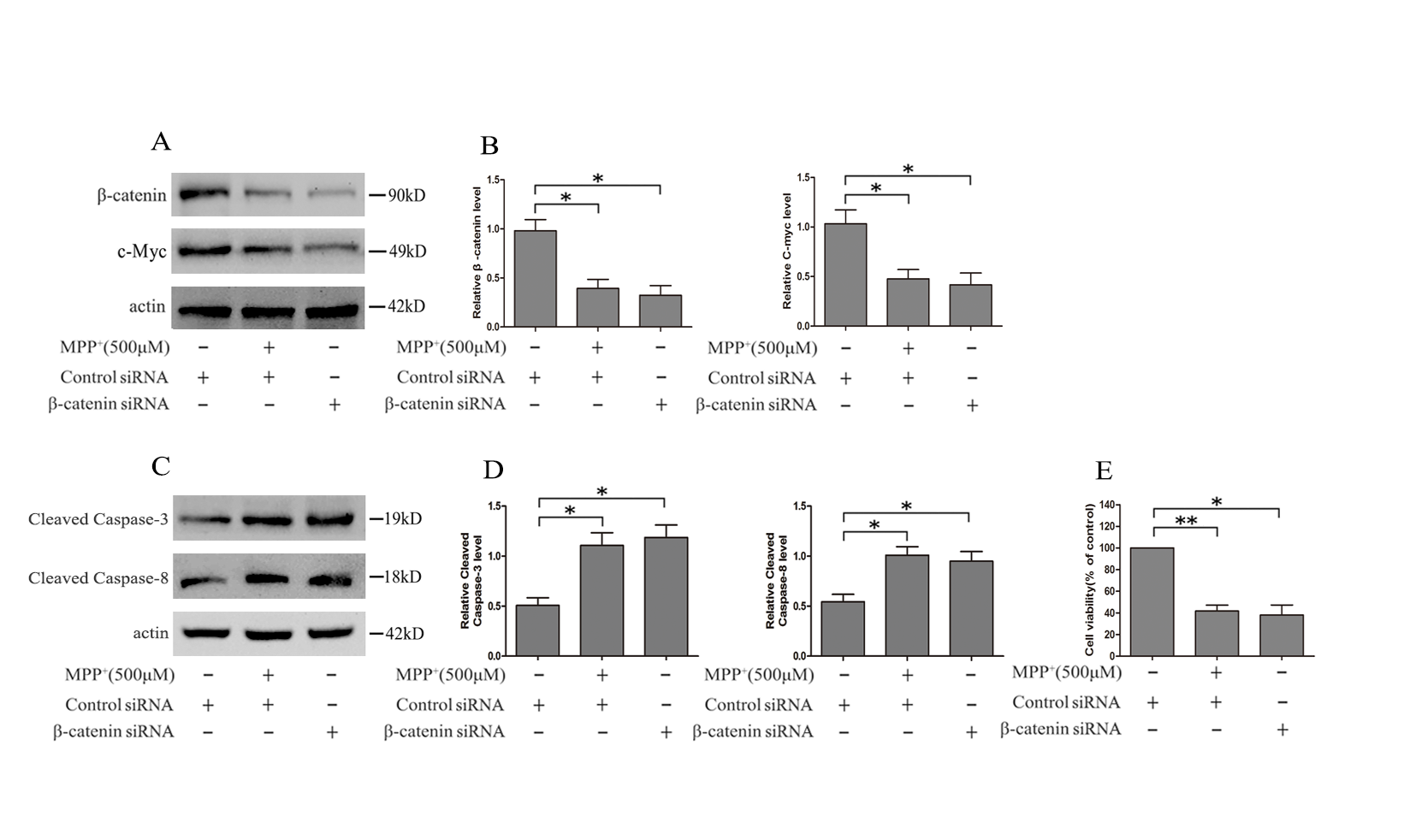


**Supplementary Figure.5:** The effects of MPP^+^ or β-catenin siRNA treatment on the expression of Wnt signaling and the process of apoptosis in SH-SY5Y cells. A: Cells transfected with control siRNA or β-catenin siRNA for 48h were then treated with MPP^+^ (500μM) for 24h. B: The ratios of β-catenin/actin and c-Myc/actin were analyzed(the means± SEM; n=3; *p<0.05). C: Cells were treated as described in A. D: The expression of cleaved-caspase-3, 8 was analyzed (the means±SEM; n=3; *p<0.05). E: The MTT assay was conducted in cells treated as described in A (the means±SEM; n=3; *p<0.05, **p<0.01).
